# TARS: A Novel Mechanism for Truly Autonomous Resource Selection in LTE-V2V Mode 4

**DOI:** 10.3390/s21227431

**Published:** 2021-11-09

**Authors:** Izaz Ahmad Khan, Syed Adeel Ali Shah, Adnan Akhunzada, Abdullah Gani, Joel J. P. C. Rodrigues

**Affiliations:** 1 Department of Computer Science and Information Technology, University of Engineering and Technology (UET), Peshawar 25000, Pakistan; azaz@bkuc.edu.pk (I.A.K.); adeel@uetpeshawar.edu.pk (S.A.A.S.); 2 Department of Computer Science, Bacha Khan University, Charsadda 24420, Pakistan; 3 Faculty of Computing and Informatics, University Malaysia Sabah, Kota Kinabalu 88400, Malaysia; adnan.akhunzada@ums.edu.my; 4 Research and Development, Senac Faculty of Ceara, Fortaleza 60160-194, Brazil; joeljr@ieee.org; 5 Instituto de Telecomunicações, 6201-001 Covilhã, Portugal

**Keywords:** LTE-V2V Mode 4, 3GPP, eNodeB, resource collision, vehicular network, road safety applications

## Abstract

Effective communication in vehicular networks depends on the scheduling of wireless channel resources. There are two types of channel resource scheduling in Release 14 of the 3GPP, i.e., (1) controlled by eNodeB and (2) a distributed scheduling carried out by every vehicle, known as Autonomous Resource Selection (ARS). The most suitable resource scheduling for vehicle safety applications is the ARS mechanism. ARS includes (a) counter selection (i.e., specifying the number of subsequent transmissions) and (b) resource reselection (specifying the reuse of the same resource after counter expiry). ARS is a decentralized approach for resource selection. Therefore, resource collisions can occur during the initial selection, where multiple vehicles might select the same resource, hence resulting in packet loss. ARS is not adaptive towards vehicle density and employs a uniform random selection probability approach for counter selection and reselection. As a result, it can prevent some vehicles from transmitting in a congested vehicular network. To this end, the paper presents Truly Autonomous Resource Selection (TARS) for vehicular networks. TARS considers resource allocation as a problem of locally detecting the selected resources at neighbor vehicles to avoid resource collisions. The paper also models the behavior of counter selection and resource block reselection on resource collisions using the Discrete Time Markov Chain (DTMC). Observation of the model is used to propose a fair policy of counter selection and resource reselection in ARS. The simulation of the proposed TARS mechanism showed better performance in terms of resource collision probability and the packet delivery ratio when compared with the LTE Mode 4 standard and with a competing approach proposed by Jianhua He et al.

## 1. Introduction

The convergence of wireless communication and vehicular networks has spurred road safety applications and use cases for safer and efficient traveling on roads [1,2]. Accurate gathering of traffic information and subsequent dissemination of the information in real time are the foundations of the road safety applications. Traffic information may include vehicle state information such as speed, direction, yaw rates, and emergency warning messages to alert the vehicles in the network.

The Wireless Access for Vehicular Environment (WAVE) standard is used to disseminate traffic information. WAVE employs a wireless communication channel, which is shared among the vehicles and infrastructure. All the vehicles are perfectly synchronized to transmit on the shared wireless channel. A vehicle must contend with and acquire the shared wireless channel before transmission. The downside of shared wireless resources in the WAVE comes from the limited channel capacity, which is subject to frequent saturation, which causes packet loss and inaccurate traffic information [3].

**Figure 1:**
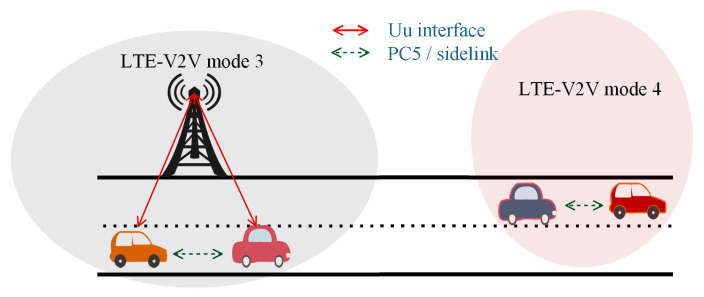
LTE-V2V Mode 3 and Mode 4.

More recently, the Long-Term Evolution (LTE)-based Vehicle-to-Everything (V2X) proposal has shown potential to improve upon the channel saturation experienced in the WAVE [4,5,6,7,8,9,10,11]. Explicitly, Release 14 of the Third Generation Partnership Project (3GPP) includes support for Vehicle-to-Vehicle (V2V) services [12,13]. There are two types of channel resource scheduling, i.e., (1) controlled by Evolved Node B (eNodeB), i.e., LTE-V2V Mode 3, and (2) a distributed scheduling carried out by every vehicle, also known as the Autonomous Resource Selection (ARS), i.e., LTE-V2V Mode 4, shown in Figure 1. Resource scheduling via eNodeB provides a centralized mechanism of allocating resources to vehicles, and it involves a slight delay before communication occurs. vehicle safety applications require more spontaneous transmission of messages; therefore, the distributed scheduling approach in Release 14 of the 3GPP is preferred for safety applications [14].

Vehicles in distributed scheduling autonomously select resources from a set of periodically reoccurring resource pools. Each vehicle then broadcasts its selection through a message called Scheduled Assignment (SA). This broadcast helps prevent other vehicles from selecting the same resource. However, resource collisions may still occur in the initial selection, where multiple vehicles can select the same resource at a time [15]. Vehicles involved in resource collisions might not cooperate with other vehicles, making the performance of the network questionable and bringing Release 14 of the 3GPP away from its design objectives for vehicle safety communication.

It is also worth noting that counter selection specifies the time for which allocated resources can be used by a vehicle. Upon expiry of the counter value, the resources must be released and reselected. The mechanism of specifying a counter value for resource usage and probability of reselecting the same resource for communication is purely based on the uniform random selection probability [12]. It is argued that the uniform random selection probability for counter selection and reselection of the same resources is an unfair approach, as it can circumvent some vehicles during transmission in congested vehicular networks. While the periodic transmission of vehicle safety messages and the broadcast nature cannot be given up, the mechanisms for resource selection can be deployed such that the collisions in ARS are reduced and the resource selection is fair towards all vehicles in congested vehicular networks.

To this end, the paper presents a Truly Autonomous Resource Selection (TARS) mechanism for vehicular networks. TARS considers resource allocation as a problem of locally detecting the selected resources at neighbor vehicles. To ensure the effect of the random uniform selection probability, TARS employs the crypto-hash function. To observe the behavior of resource selection and collision, this paper presents a Discrete Time Markov Chain (DTMC) model for the counter selection and resource reselection. Subsequently, insights from the model are used to specify the mechanism for fair counter selection, as well as the resource reselection policy.

The rest of the paper is divided into the following sections. In Section 2, an overview of the related work is presented. Preliminaries regarding the Device-to-Device (D2D) communication are given in Section 3. Section 4 explains the system model and proposed TARS mechanism. The performance of the proposed technique is evaluated in Section 5. Lastly, the paper is concluded in Section 6.

## 2. Related Work

A substantial research effort has been conducted aiming at efficient resource allocation for LTE-based vehicular communication. A two-stage distributed resource allocation technique was presented in [16] to assist the outside network coverage scenario of LTE-based V2V communication. Initially, vehicles are classified in subpools according to their orthogonal directions, followed by Resource Block (*RB*) assignment based on the directions in which they are moving. Automobiles traveling in the same direction are granted the same set of resources. Secondly, in order to avoid packet collision between vehicles traveling parallel to each other, a channel-sensing mechanism is employed. The proposed scheme provides better Collision Avoidance (CA) for ARS mode of resource allocation in LTE.

The authors in [14] proposed a collision-avoidance scheme for ARS in vehicular communication based on LTE. The performance of ARS is significantly improved by the proposed algorithm. To inform the neighbor vehicles about the reserved data packets, SA information is piggybacked on the data packets in the reservation phase, resulting in improved reliability and minimized data packet collisions.

In [17], an optimized resource allocation for LTE-V2X networks was achieved, keeping different sizes of periodic broadcast packets in consideration. The number of automobiles that can be assigned when the parameters are upgraded for messages of various extents was valuated. The effect of some transmission parameters on the count of vehicles was examined.

The authors presented an improved resource selection approach in [18] for V2V communication, which delivers an ARS grounded on a defined frame structure, involving SA and data transmission. Sensing results are utilized to mitigate collisions in a secondary approach. The proposed mechanism is analogous to the LTE Release 12 side link communication with reference to throughput and the probability of collision.

A novel collective ARS and scheduled resource assignment mechanism was introduced in [19]. The goal is to augment the aggregate of the data values of users. The presented approach takes the user’s Proximity Service (ProSe) Per-Packet Priority (PPPP) and the reliability of the communication into account, while satisfying the Quality of Service (QoS) of Vehicular User Equipment (V-UE) and Pedestrian User Equipment (P-UE).

The authors presented a D2D resource allocation and power control (DRAPC) strategy for sharing spectrum and allocating power for D2D communications in [20]. There were four resource sharing models proposed, in which the Cellular User Equipment (CUE) and Machine-to-Machine (M2M) pairings were related to one another in one-to-one, one-to-many, many-to-one, and many-to-many ways. M2M communication minimizes the network traffic burden because the communication does not pass through eNodeB.

Radio Resource Management (RRM) for V2X was presented in [21], which offers reliability and latency when assigning resources to vehicles and is based on D2D communication. To ensure the sum-rate and reliability constraints, the authors explored a unicast communication paradigm. In a highway scenario, the signal power and buffer size were chosen as the key parameters for V2X communication.

To improve the network’s performance, an algorithm for relay-based two-hop D2D communication was developed in [22]. A resource allocation approach with minimal interference was presented, which can be used in cellular networks with two-hop D2D communications. eNodeB assigns cellular users’ resources to D2D users, based on signal-to-interference-plus-noise ratio (SINR) with the aim of increasing system throughput.

In [23], the authors presented a method for jointly optimizing relay selection, power control, and spectrum allocation. The technique involves a refined jointly optimized relay selection problem, while taking care of the constraints of EE. To reduce power consumption, the problem is represented as a bipartite graph between the UEs and a set of relays. The aim was to maximize energy efficiency while maintaining the QoS.

To obtain a greater throughput in D2D communication, the joint scheduling problem on mode selection, radio resource allocation, and power coordination was investigated in [24]. The aim was to improve the traffic offloading capacity of cellular-assisted D2D communication by incorporating D2D relays. Relay-aided and “local route” (i.e., devices communicate utilizing the BS as a relay station) D2D were evaluated, and the suggested model increased the capacity of relay-enabled cellular-assisted D2D communication. The simulation results indicated that the suggested technique improves the traffic offloading capacity of D2D-capable UEs in LTE networks.

## 3. Preliminaries of D2D Communications

### 3.1. D2D Communication

The 3GPP is developing an enhanced LTE radio interface called LTE-Advanced (LTE-A) to overcome the shortcomings of LTE. The LTE-A radio interface has many features including D2D communication, Multiple-Input Multiple-Output (MIMO), carrier aggregation, and millimeter wave. D2D communication is a direct communication amongst two closely located UE without passing over the base station (eNodeB/eNB) or the central network Evolved Packet Core (EPC). The communication occurs on a Side Link (SL) or PC5 interface between the two devices [25]. D2D communication promises three kinds of benefits: (1) the proximity of UE; this permits enhanced bit rates, lower delays, and less power consumption; (2) reuse gain; radio resources can be used by cellular and D2D links at the same time; (3) hop gain; there is one link in the D2D mode instead of the Uplink (UL) and Downlink (DL) when communicating through eNodeB [26].

**Figure 2:**
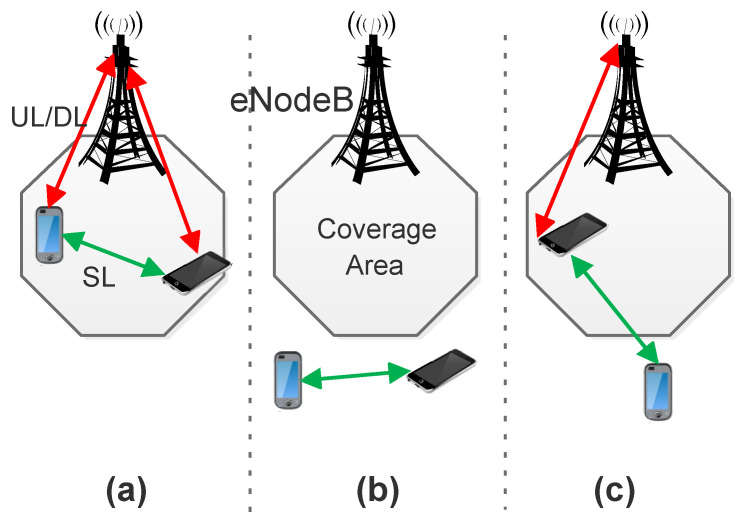
D2D communication scenarios: (**a**) within network coverage, (**b**) outside network coverage, and (**c**) partial network coverage.

Based on the coverage of the LTE network, D2D communication can occur in three ways: within network coverage, outside network coverage, and partial network coverage. In the partial network coverage scenario, the in-coverage UE acts as a relay between the out-of-coverage UE and eNodeB, resulting in an increase in the coverage area of eNodeB [27]. Figure 2a,b and c shows the communication scenarios for D2D.

Release 14 of 3GPP reveals that vehicles can communicate with other vehicles based on the principles of D2D communication. Figure 2b represents Mode 4 communication in Release 14, wherein vehicles can manage and select resources from available resources without the involvement of the core cellular network [12].

### 3.2. LTE-V2V Mode 4

Mode 4 is studied as the baseline for V2V communication because of its ability to operate without the involvement of cellular infrastructure. Safety applications based on V2V communication cannot depend on the coverage of cellular network, making Mode 4 even more suitable for such communication. Vehicles in Mode 4 use PC5/the side link for V2V communication. Radio resources are autonomously selected, having no dependency on cellular network coverage. For in-coverage vehicles, the configuration decision of the V2X channel is performed and sent to vehicles by the network through the PC5 V2X configurable parameters. The message comprises the V2X resource pool, the number of subchannels in a subframe, the number of *RB*s in a subchannel, the carrier frequency, etc. [28].

For vehicles present outside network coverage, a set of preconfigured parameters is used to replace the configurable PC5 V2X parameters. However, an exact value of each parameter is not specified in the standard. The subframes utilized for V2X communication are indicated in the V2X resource pool. Remaining subframes can be exploited for cellular communication. Geographical-area-based division of the V2X resource pool is included in the standard termed zoning. Vehicles in a specific area can use the resources assigned to that area [28]. In this paper, the channel was assumed to be entirely dedicated to V2X communication.

In LTE Mode 4, resources are arranged as blocks called *RB*s. The following section discusses the mechanisms used for the selection of these blocks.

### 3.3. Resources in LTE-V2V Mode 4

#### 3.3.1. Anatomy of RB in Mode 4

**Figure 3:**
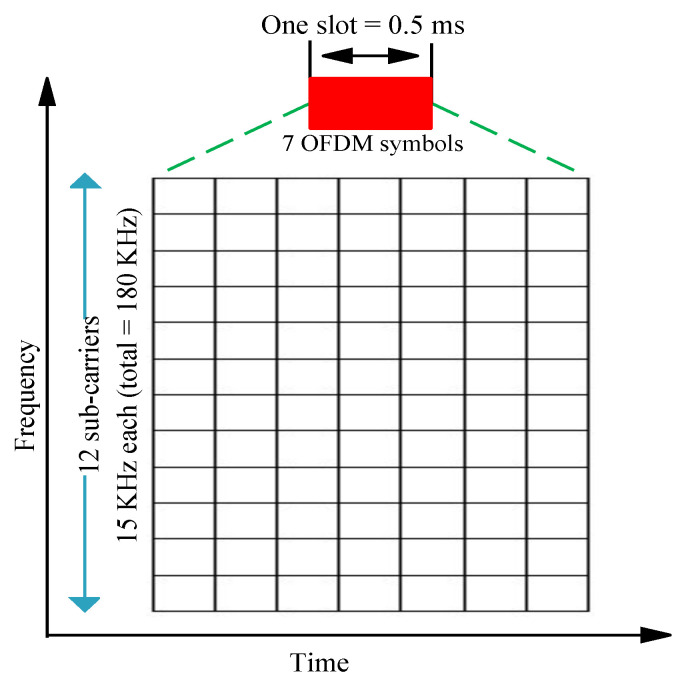
A Resource Block (*RB*).

The smallest block of resources represented in the time and frequency domain is referred to as an *RB*. *RB*s are used for DL/UL transmission. A group of 12 contiguous subcarriers, having a subcarrier spacing of 15 kHz, over a single time slot comprise an *RB*, as shown in Figure 3.

One *RB* corresponds to 180 kHz (i.e., 15 × 12 = 180) in the frequency domain over one slot (1 slot = 0.5 ms). There are seven Orthogonal Frequency Division Multiplexing (OFDM) symbols in each time slot [29]. Transmissions are allotted in units of *RB*s. In [30], the number of *RB*s corresponding to their respective channel bandwidths was presented, i.e., channels with bandwidths of 1.4 MHz, 3 MHz, 5 MHz, 10 MHz, 15 MHz, and 20 MHz correspond to 6 *RB*s, 15 *RB*s, 25 *RB*s, 50 *RB*s, 75 *RB*s, and 100 *RB*s, respectively.

#### 3.3.2. Resource Selection

LTE-enabled devices/vehicles require wireless resources to broadcast messages. Two mechanisms of resource (*RB*s) scheduling defined in Release 14 of the 3GPP are: (a) Scheduled Resource Allocation (SRA), referred to as Mode 3, and (b) ARS, referred to as Mode 4 [12].

**Figure 4:**
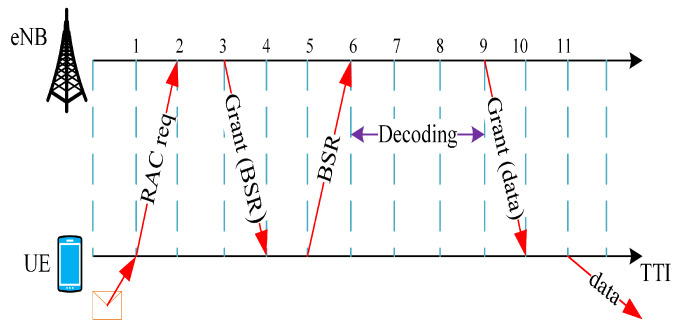
Resource allocation in SRA, Mode 3.

SRA is a centralized mechanism wherein a UE initially sends a Radio Access Control (RAC) request to the associated eNodeB, that in return allots some resources for it to send the status of its buffer via a Buffer Status Report (BSR). After this report, eNodeB schedules resources to the LTE UE for D2D communication [31], as illustrated in Figure 4.

Due to the signaling overhead involved for each UE, Mode 3 is not a good choice for delay-critical applications.

**Figure 5:**
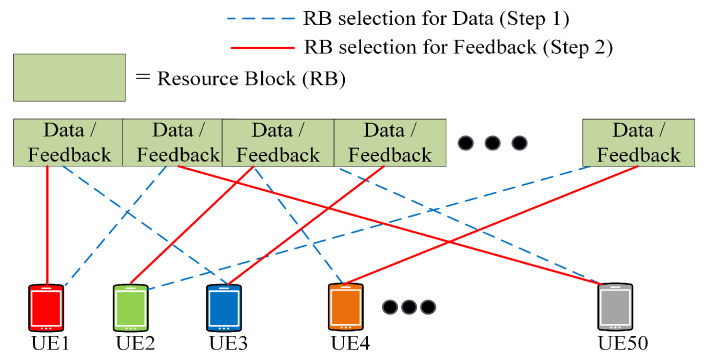
Resource selection in ARS, Mode 4.

ARS uses a distributed approach. A static resource pool comprising many *RB*s is presented after every Transmission Time Interval (TTI). The UE can autonomously select *RB*s from it with no prior signaling [31]. Figure 5 presents this idea. The dotted line in Figure 5 represents *RB* selection for data transmission. Step 2 involves sending feedback regarding *RB* selection.

Mode 4 does not involve signaling for the request of resources from LTE, and hence, it is more suitable for delay-constrained vehicle safety applications. However, the lack of signaling also means contention for the *RB* selection and the probability of resource collisions [32]. The probability of resource collisions is bound to increase for densely populated networks [16]. This scenario of resource collisions among multiple vehicles and the broadcast nature of vehicle safety messages brings Release 14 of the 3GPP away from its design objectives of LTE-V2X for vehicular broadcast communications.

## 4. System Model Assumptions and Proposed Mechanism

### 4.1. System Description

To understand the behavior of resource collisions, a discrete-time ARS mechanism is considered. Vehicles having data to transmit must select *RB*s from a periodically reappearing pool of *RB*s. Subsequently, the selected *RB*s are used to transmit the data. According to the standard [33], the model considers three processes, i.e., (1) *RB* selection, (2) counter selection, and (3) *RB* reselection. When a vehicle has data to transmit, the *RB* selection process executes, which refers to the initial selection of *RB*s from a pool of resources. Upon the selection of the *RB*, the counter selection process starts, which refers to the selection of an integer counter value. This counter defines the number of times the selected *RB* can be used for subsequent transmissions. Finally, upon expiry of the counter, the *RB* reselection process is called, which refers to the probability of reselecting the same *RB* for further transmissions. These three processes are called *Process 1*, *Process 2*, and *Process 3*, respectively.

### 4.2. System Model

To observe the behavior of *RB* selection and collision, it is important to model *Process 1*, *Process 2*, and *Process 3*. The probability of *RB* collision in the context of *Process 1* in ARS is elementary. For *n* users and a number of *RB*s *r*, *RB* selection is an independent event of *RB* selection from a single pool, performed by every vehicle, and it is given by n/n(n−r)!. Therefore, the system model only considers *Process 2* and *Process 3*. Symbols/acronyms and their abbreviations are listed in Table 1.

Considering *Process 2* and *Process 3* as the DTMC, the following assumptions were made:

- If a user has data to transmit, then the user has already selected an *RB* for the transmission of the data; hence, *Process 1* is omitted;
- The number of transmitting users is directly proportional to the *RB* collisions;
- A user is considered as a transmitting user only after reselecting the same resource for further transmission. The transmitting users are the ones that have more data, and they contend for more resources, hence resulting in more collisions.

Therefore, to observe the behavior of *Process 2* and *Process 3* in terms of the number of transmitting users, let $X_{n}$ be the number of transmitting users in ARS at any instant of time,

(1) $$X_{n}\;\in \;S\;:=\left\{0,1,2,\ldots ,N\right\}$$

where *X* represents the ARS process, *S* is the state space, *n* is the time slot, and *N* is the total number of transmitting users.

A user that has data to transmit always enters *Process 2* to select a counter. The probability of *Process 2* is defined by the probability that a user has data to transmit. Therefore, the probability that a user has data to transmit and that it is in *Process 2* in time slot *n* is given by γ. It was assumed that after *Process 2*, a simultaneous event also occurs, i.e., *X* enters in *Process 3* at time *n*. Then, the probability of reselecting the same *RB* is given by δ, and the probability of not selecting the same *RB* is given by (1−δ). Once a counter and reselection counter are selected, the user is considered as a transmitting user at time slot n+1.

The state transitions in *X* happen when *X* enters in *Process 3*. When a user has data to transmit at time *n*, the number of transmitting nodes $X_{n}$ either increases or decreases at n+1 depending on the probabilities of *Process 2* and *Process 3*. The transition probability matrix *P* is given by:

(2) $$P_{x,t}=\left\{\begin{array}{cc} p_{1}=\gamma \left(\delta \right), & \left\{t=x+1|x=1,2,3,\ldots \right\}\\ p_{2}=\gamma \left(1-\delta \right), & \left\{t=x-1|x=1,2,3,\ldots \right\}\\ p_{3}=\left(1-\gamma \right)\left(1-\delta \right), & \left\{t=x|x=1,2,3,\ldots \right\}\\ \gamma , & \left\{x=0,t=1\right\}\\ (1-\gamma ), & \left\{x=0,t=0\right\} \end{array}\right\}$$

where $P_{x,t}$ is the transition probability matrix from the current state *x* to the next state *t*. The transitions happen when the user enters *Process 2* and *Process 3*. Observe that, at *n*, if γ and δ are selected, then $X_{n}$ increases by one, i.e., $X_{n+1}=X_{n}+1$. $X_{n}$ decreases if, after counter selection, the same *RB* is not selected. Similarly, $X_{n+1}=X_{n}$, if the user has no data to transmit and *Process 2* and *Process 3* are not called.

**Figure 6:**
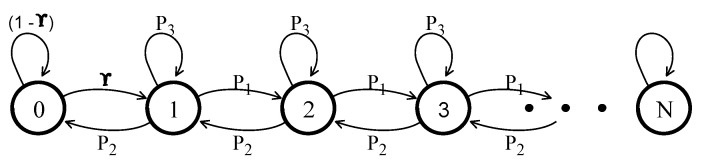
Birth and death Markov chain.

Observe that the DTMC is very similar to the discrete time birth and death Markov chain, as shown in Figure 6. Therefore, the balanced equations of $X_{n}$ are:

(3) $$\pi_{0}*\gamma =\pi_{1}*p_{2}$$

where $\pi_{i}$ is the frequency of being in a certain state. Similarly, according to Figure 6:

(4) $$\pi_{1}=\pi_{0}*\gamma +\pi_{1}*p_{3}+\pi_{2}*p_{2}$$

We can further generalize the equations for state *n*, where n≥2, as follows:

(5) $$\pi_{n}=\pi_{n-1}*p_{1}+\pi_{n}*p_{3}+\pi_{n+1}*p_{2}$$

(6) $$\pi_{n}=\sum_{m=0}^{x}\left(p_{i,n}\pi_{m}\right)$$

**Figure 7:**
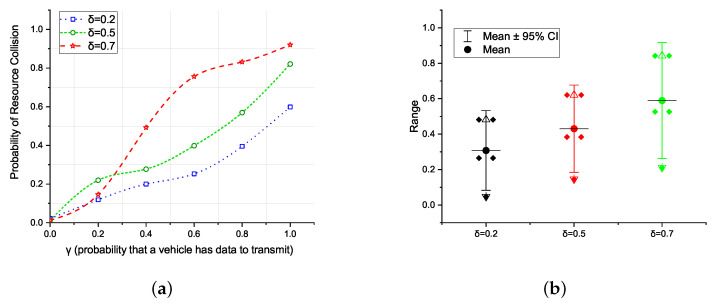
Resource collision probability; (**a**) of the Markov model and, (**b**) a confidence interval of 95%.

The collision probability is plotted in Figure 7a. The X-axis shows the probability of a node having data to transmit, i.e., γ. The Y-axis shows the resource collision probability. In addition, the degree of uncertainty is analyzed at a confidence interval level of 95% to show the range in which the values of & = 0.2, & = 0.5, and & = 0.7 fall.

The plotted graph shows the collision probability for different values of δ. Observe that for higher values of δ and γ, the collision probability increases rapidly. The behavior of the DTMC suggests that the design of the ARS mechanism should be adaptive towards the vehicle density and/or traffic intensity to reduce resource collisions. In the following section, we present the TARS mechanism, which not only employs a novel technique for autonomous resource selection, but also is adaptive towards the vehicle densities.

### 4.3. Proposed Approach

Enhancement in collision avoidance for the LTE D2D side link resource allocation provides significant benefits for LTE broadcasting. For the selection of *RB*s, our proposed approach utilizes the crypto-hash function. Through the use of the crypto-hash function, the aim was to achieve the following two tasks: (1) to provide randomness in the selection of an *RB* by exploiting the avalanche effect and (2) to predict the resource selection of the neighbors to a certain level through the use of the input to the crypto-hash.

The avalanche effect is the property of algorithms for which, for a slight change in the input, there is a significant change in the output (almost half the bits flip) [34]. The proposed approach exploits this property in Message Digest 5 (MD5), which has a 128 bit hash value expressed as a 32-digit hexadecimal number. In addition, by handpicking the seed for the crypto-hash function, the *RB* selection of the neighbor vehicles can be predicted to a certain level.

### 4.4. TARS Mechanism

**Figure 8:**
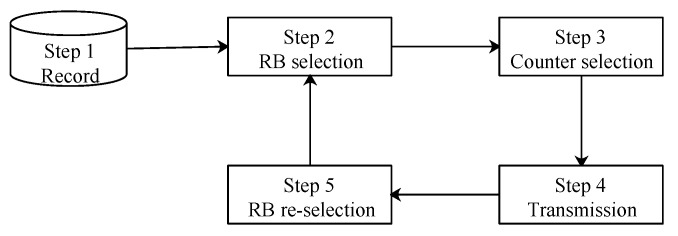
Workflow of TARS.

Truly Autonomous Resource Selection (TARS) has an iterative process including (a) *RB* selection, (b) counter selection, (c) the transmission of the message, and (d) *RB* reselection, as depicted in Figure 8.

The following steps are involved in TARS.

#### 4.4.1. *RB* Selection

The purpose of *RB* selection in TARS is to ensure that neighbor vehicles select unique *RB*s to avoid resource collision. To control resource collisions effectively, the neighbors’ *RB* information is locally available on every vehicle, and there is a local mechanism to predict the selected *RB*s by the neighbors as well.

The neighbors’ information is calculated by every vehicle by monitoring the signal strengths of the *RB*s. This information includes the neighbor identification and the corresponding resource records. This information is gathered after every 5 s interval for every vehicle. In addition, the stored record is also weighted in such a way that the recently selected *RB*s have a higher weight than the old *RB*s.

(7) $$R_{s}=\sum_{j=1/10}^{1}\sum_{i=1}^{n}w_{j}\;R_{i}\;,\;w_{1}<w_{j-1}<w_{j}$$

where $R_{s}$ is the selected *RB*, *w* is the weighted moving average, $w_{1}$ represents the oldest recorded value, and $w_{j}$ is the latest value. Preference is given to the latest value. $R_{i}$ is the set of *RB*s.

**Figure 9:**
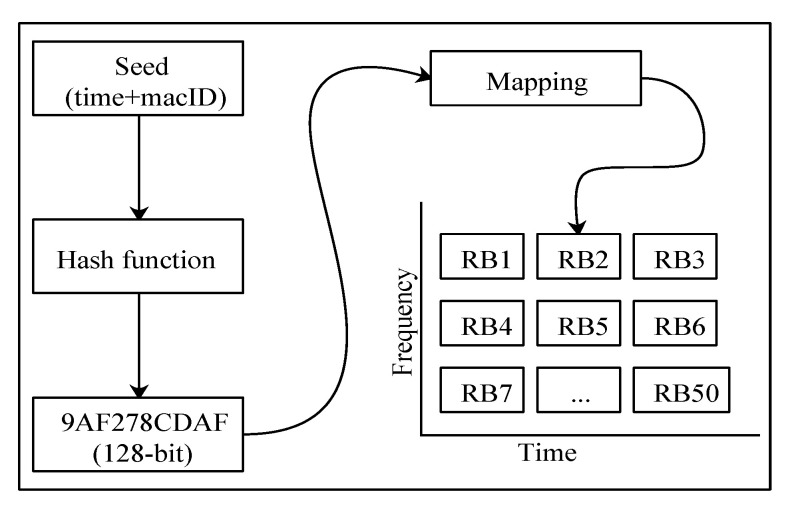
*RB* selection.

Once the neighbor information is populated on every vehicle, TARS uses a crypto-hash function to select an *RB* for transmission by predicting the selected *RB*s of its neighbors. Explicitly, the neighbor information, along with time as a random variable, is fed into the crypto-hash function to acquire a 128 bit value. The 128 bit value is then pruned and mapped to one of the 50 *RB*s using Equation (8), as shown in Figure 9. Each vehicle calculates *RB*s in the same way for all of its neighbors.

(8) $$RB=\left(val_{cur}-val_{min}\right)*\frac{50}{\left(val_{max}-val_{min}\right)}$$

where $val_{cur}$ is the decimal-converted equivalent of the last two hexadecimal digits obtained by using MD5 hash. The number 50 shows the considered range of *RB*s. $Val_{max}$ and $val_{min}$ shows the maximum and minimum decimal equivalents for two hexadecimal digits, i.e., FF = 255 ($val_{max}$) and 00 = 0 ($val_{min}$).

#### 4.4.2. Counter Selection

According to the standard [33], once an *RB* is assigned to a vehicle, a selection counter is randomly set up between five and fifteen. The counter is decremented by one after each packet transmission. For congested scenarios, the selection of a counter value with a uniform random selection probability has limitations. For instance, a vehicle in a congested network and in a scarce network has equal probability of selecting a higher counter value and vice versa. We argue that the counter selection should be adaptive and based on the network congestion. This ensures that vehicles in a congested network autoselect lower counter values to allow fair opportunities for transmission for neighbor vehicles.

To incorporate adaptiveness in counter selection, the proposed approach divides the selection counter window into two, i.e., [5 to 10] and [10 to 15]. Subsequently, Equation (9) is used to find the probability of setting the counter between the two selection windows.

(9) $$S_{c}=1-\left[R_{s}^{-1}*\beta \right]\;,\;\beta \to 35\%$$

where $S_{c}$ is the selection counter, $R_{s}^{-1}$ is the inverse of channel load, and β is a threshold after which our selection counter is used. As a design choice, β is set to 35%. The result of Equation (9) is the probability for setting the counter between [5 to 10], and (1 – $S_{c}$) is the probability for setting the counter between [10 to 15]. For $R_{s}^{-1}$ less than β, our approach follows the standard, i.e., the counter is set between [5 to 15] and for $R_{s}^{-1}$ greater than or equal to β, and when the channel is busier, Equation (9) is used, i.e.,

(10) $$\left\{\begin{array}{cc} R_{s}^{-1}<\beta , & S_{c}\left(Std\right)\\ R_{s}^{-1}\geq \beta , & S_{c} \end{array}\right.$$

**Figure 10:**
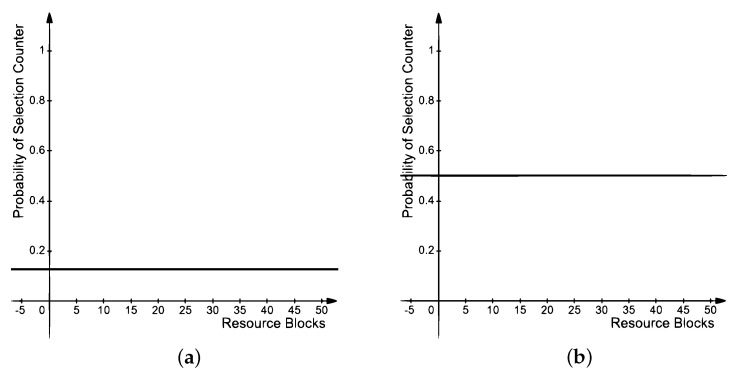
Probabilities of the proposed selection counter; (**a**) For $R_{s}$ = 0.4, (**b**) For $R_{s}=0.7$.

When the channel is busy, the proposed approach sets the counter to a smaller value. The significance of defining two ranges for the counter and adaptive counter value selection is bound to provide fairness in channel access in congested scenarios. Figure 10a,b shows the effect of using $S_{c}$ for counter selection.

#### 4.4.3. *RB* Reselection

**Figure 11:**
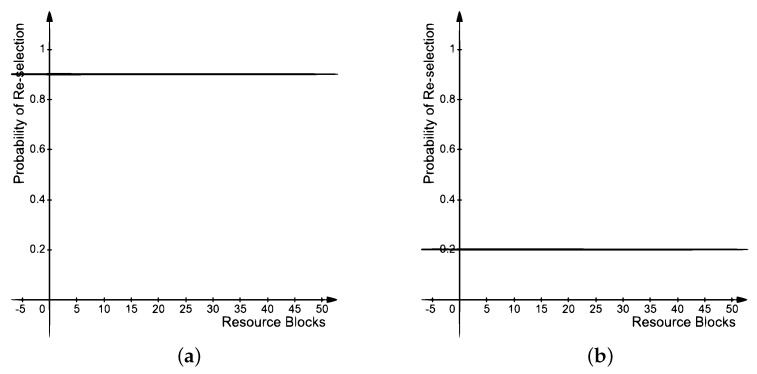
Probabilities of reselection; (**a**) For $R_{x}$ = 5, (**b**) For $R_{x}$ = 40.

It is known from the standard that after the start of transmission, once the selection counter reaches zero, the reselection of resources must be performed with probability (1−p), where the value of *p* is set between [0–0.8] [33]. This can cause unfair selection for dense networks. As an example, a vehicle with a lower value of *p* may cause some vehicles to wait for their turn for *RB* selection. The value of *p* should be inversely proportional to the network density and not set as a random value between [0–0.8].

(11) $$P_{r}=1-\left(\frac{\forall \;R_{x}\;\in \;R_{i}|R_{x}\geq \tau }{R_{i}}\right)$$

where $P_{r}$ is the probability of reselecting the same *RB*, τ is the threshold, which is set to less than or equal to 20% as in the standard [33], and $R_{x}$ is some *RB*s out of the total *RB*s ($R_{i}$). $R_{x}$ ≥ τ means the set of $R_{x}$ greater than or equal to 20% free. Figure 11a,b shows the effect of using $P_{r}$ for *RB* reselection.

#### 4.4.4. Algorithm

This section describes the TARS algorithm. The inputs for Algorithm 1 include local neighbor information $l_{n}$, current local time $t_{l}$, and channel information in terms of the signal-to-noise ratio $ch{\left(i\right)}_{SNR}$. A successful algorithm execution gives the resource block, $RB_{l}$, to be used by a vehicle for beacon transmission.

Initially, a vehicle needs $l_{n}$, $t_{l}$, and $ch{\left(i\right)}_{SNR}$. $l_{n}$ is acquired through the received beacons from the neighbors and stored in a list. This information contains the unique MAC identifier for a neighbor. It was assumed that the time at every vehicle is perfectly synchronized. This is a reasonable assumption because of the prevalent time synchronization standards. $ch{\left(i\right)}_{SNR}$ is the signal-to-noise ratio measurement of all the *RB*s. This information is stored in a table where every *RB* is recorded, along with its previous SNR value.

In Lines 6–7, if a vehicle has a beacon to transmit, it first selects a suitable *RB*. The algorithm uses a 64 bit crypto-hash function, which is pruned and mapped to an *RB* number. From Line 8 to Line 10, to avoid resource collision with neighbors, each vehicle tries to predict the *RB* selection by its neighbors. For each of its neighbors, a vehicle uses a seed that includes the MAC address of a neighbor and $t_{l}$ to acquire a list of all the *RB*s selected by its neighbors. In Line 12 and Line 13, if the vehicle finds resource collision, then the vehicle selects an *RB* that has the least SNR value across all the *RB*s.

From Lines 15–17, the process of counter selection is called. The value of the counter is determined via Line 22–25. If the value of β is less than 35%, then the standard value of counter $S_{c}$ (Std) is used, which is [5–15]; otherwise, Equation (9) is used to set up the counter value. At Line 27, transmission is performed on the selected *RB*. *RB* reselection is carried out in Lines 30–34 when the counter value reaches zero. If the same *RB* is reassigned, transmission is established; otherwise, the process of acquiring a new *RB* is restarted via *RB* selection.
**Algorithm 1***RB* selection using TARS.**inputs:**$l_{n},t_{l},ch{\left(i\right)}_{SNR}$**output: $RB_{l}$**1:**procedure** Main2:    **procedure** *RB*Selection3:        $Get\;Neighbor\;list\leftarrow l_{n}$4:        $Get\;GNSS\leftarrow t_{l}$5:        $Get\;SNR_{c}{}_{h}{}_{(}{}_{i}{}_{)}\leftarrow ch{\left(i\right)}_{SNR}$6:        $Calculate\;hash_{l}\leftarrow \left(l_{n}+t_{l}\right)$7:        $Map\;hash_{l}\leftarrow RB_{l}\;using\;Equation\;\left(8\right)$8:        **for** $\left(each\;i\;in\;ch{\left(i\right)}_{SNR}\geq 20\%\right)$ **do**9:              $Calculate\;hash_{l}\leftarrow \left(l_{i}+t_{l}\right)$10:            $Map\;hash_{l}\leftarrow RB_{i}$11:       **end for**12:       **for** $\left(each\;i\;in\;ch{\left(i\right)}_{SNR}\right)$ **do**13:             **if** $\left(RB_{l}==RB_{i}\right)$ **then**14:                   $Select\;RB_{l}\;ch{\left(i\right)}_{SNR}:ch{\left(i\right)}_{SNR}<\forall \;ch{\left(i\right)}_{SNR}$15:                   CounterSelection()16:             **else**17:                   CounterSelection()18:              **end if**19:        **end for**20:    **end procedure**21:    **procedure** CounterSelection22:         **if** (β<35%) **then**23:               $counter==S_{c}\left(Std\right)$24:         **else**25:                counter←usingEquation()26:         **end if**27:         $Transmit\;using\;RB_{l}$28:    **end procedure**29:    **procedure** ReSelection30:         RBreslection←usingEquation()31:         **if** $\left(RB_{n}{}_{e}{}_{w}==RB_{l}\right)$ **then**32:                  $Transmit\;using\;RB_{l}$33:         **else**34:                  RBselection()35:         **end if**36:     **end procedure**37:**end procedure**

## 5. Performance Evaluation

The model for TARS was implemented in MATLAB, and its performance was compared with the LTE Mode 4 standard and a competing autonomous resource selection approach proposed and described in [14]. The simulation parameters are given in Table 2.

**Figure 12:**
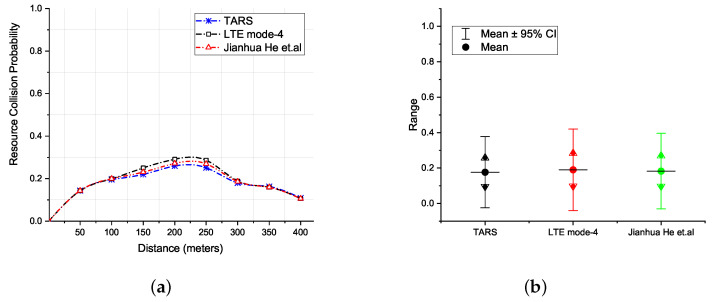
Resource collision probability; (**a**) with respect to distance and, (**b**) a confidence interval of 95%.

The result in Figure 12a shows the resource collision probability with respect to increasing distances. The probabilistic range of values for collision probability is also depicted in the figure at a confidence interval level of 95%. At lower distances, the resource collision probability is negligible. As the distance between the sender and receiver increases, i.e., beyond 50 m, the collision probability starts to increase. This is due to the increase in the number of neighbors of a vehicle, which results in higher contention for resource blocks. At even higher distances, i.e., around 250 m, the collision probability starts to reduce. Observe that at higher distances, the vehicle density remains the same and so does the transmit power for sender. Therefore, the resource collision probability reduces for higher distances. Observe that the standard, i.e., LTE Mode 4, is comparatively more prone to collisions due to the random nature of resource selection. The probability of resource collisions was reduced by He et al. due to the piggyback mechanism through SA, which contains information about the reserved *RB*s for future transmissions. However, continuous collisions are still possible during the initial selection of *RB*s from multiple vehicles. TARS, on the other hand, reduces resource collisions by effectively predicting the *RB*s being used by the neighbors, hence reducing the probability of collision.

**Figure 13:**
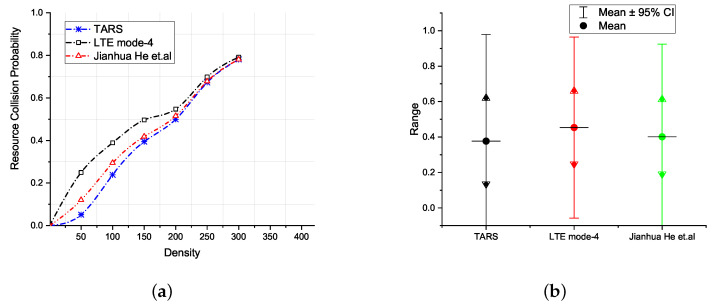
Resource collision probability; (**a**) with respect to density and, (**b**) a confidence interval of 95%.

Figure 13a reports the increase in the probability of resource collision with the increase in vehicle density and the probabilistic range of values for collision probability at a confidence interval level of 95%. Note that the increase in the probability of resource collisions with respect to density in LTE Mode 4 and He et al. is due to the uniform resource pool and the random selection of *RB*s by vehicles. TARS improves this performance through its ability to select unique *RB*s instead of a uniform random resource selection mechanism. Moreover, at lower densities, better TARS performance is due to the unique selection of resources by TARS instead of the uniform random resource selection mechanism of LTE Mode 4 and He et al. However, the at around 300 vehicles and beyond, the performance of TARS is comparable with LTE Mode 4 and He et al. This is because at higher vehicle densities, a unique selection of resources by TARS is not fully applicable because of the limited number of *RB*s.

**Figure 14:**
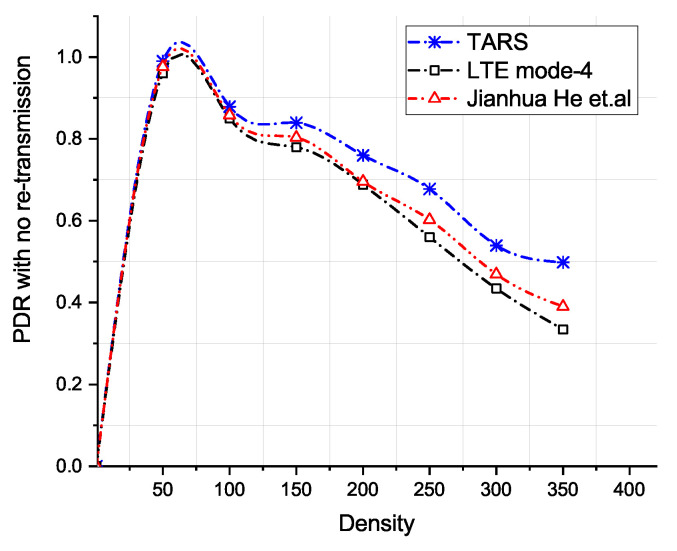
PDR with no retransmission versus density.

**Figure 15:**
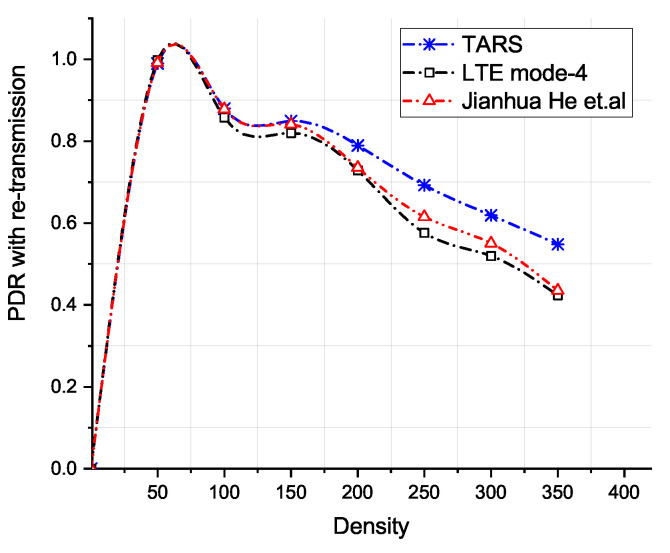
PDR with retransmission versus density.

The Packet Delivery Ratio (PDR) against increasing vehicle densities is depicted in Figure 14 and Figure 15. At lower densities, the PDR is high. It drops with the increase in the number of vehicles in the network because of the interference caused by other vehicles. We also evaluated TARS by employing retransmission mechanism as proposed in LTE Mode 4 and in He et al. The PDR of all approaches improves when packets are retransmitted. There are more *RB*s available for selection; therefore, TARS without retransmission achieves a better PDR than LTE Mode 4 and He et al. Similarly, with retransmissions, the better PDR in TARS is due to the fewer resource collisions owing to its effective local RB prediction and subsequent selection for transmission.

LTE Mode 4 and He et al. employ retransmissions for redundancy and reliability in terms of PDR. However, this has a tradeoff of consuming more bandwidth [35]. Since TARS does not rely on retransmission, TARS can also be theoretically evaluated to understand its bandwidth savings. By way of an example, the periodic message size is between 50 B and 300 B if no cryptography codes are used. Vehicle safety applications have a typical message frequency of 5 Hz to 50 Hz. If we assume a 10 ms scheduling period and one *RB* for every transmission, then in a 10 MHz bandwidth, 2 dB of noise, and 28 dBm of signal power, we are left with approximately 19 Mbps of savings in bandwidth.

## 6. Conclusions

This paper presented TARS, a resource selection mechanism that identifies resource collisions locally using a crypto-hash function. TARS locally calculates adaptable metrics for counter selection and the resource reselection probability with respect to vehicle density. The simulation results justified the importance of TARS in reducing resource collisions and enhancing the PDR with respect to different vehicle densities and distances. This verifies that resource collisions can be avoided by using a local collision identification approach. Adapting the counter selection and resource reselection with respect to vehicle density provides a fair opportunity for vehicles to transmit.

## Figures and Tables

**Table 1:** List of acronyms/symbols.

Acronyms	Abbreviations
*X*	represents the ARS process
*n*	time slot
$X_{n}$	number of transmitting users at *n*
*S*	state space
*N*	total number of transmitting users
γ	the probability that a user has data to transmit at *n*
δ	the probability of reselecting the same *RB*
*P*	transition probability matrix
*x*	current state
*t*	next state
$P_{x,t}$	transition probability matrix from *x* to *t*
$p_{1}$	the probability of moving from the current state to the next state
$p_{2}$	the probability of moving back to the previous state
$p_{3}$	the probability of staying in the same state
$\pi_{i}$	the frequency of being in a certain state

**Table 2:** Parameters.

Parameters	Values
Vehicular Density	50 to 350
Sender/Receiver Distance	50 m to 400 m
Vehicular Speed	55 km/h
No. of lanes	2 lane highway
*RB* Bandwidth	10 MHz
*RB*s	50
Tx Power	26 dBm
Pkt Size	350 B

## Data Availability

Not Applicable.
